# Air Embolism: A Rare Lethal Complication of Hysteroscopy in a Young Woman Undergoing Infertility Workup

**DOI:** 10.7759/cureus.45069

**Published:** 2023-09-11

**Authors:** Apoorva Dave, Pranjal Kashiv, Kamlesh Chaudhari, Deepti Shrivastava

**Affiliations:** 1 Obstetrics and Gynaecology, Jawaharlal Nehru Medical College, Datta Meghe Institute of Higher Education and Research, Wardha, IND; 2 Nephrology, Jawaharlal Nehru Medical College, Datta Meghe institute of Higher Education and Research, Wardha, IND

**Keywords:** endoscopy, infertility work up, complications, diagnostic hysteroscopy, air embolism

## Abstract

Compared to operative hysteroscopy, diagnostic hysteroscopy rarely leads to issues. However, one very uncommon yet potentially fatal complication is air embolism, with an incidence rate of three in 17,000 cases. This report describes an unexpected complication discovered during diagnostic hysteroscopy surgery. In the course of routine infertility testing, a 29-year-old woman underwent a diagnostic hysteroscopy under general anesthesia. Intraoperatively, her end-tidal carbon dioxide (EtCO2) levels decreased, oxygen saturation dropped, and heart rate increased, leading the anesthesiologists and critical care team to terminate the procedure and manage her further. Subsequent transesophageal echocardiography confirmed the diagnosis of air embolism. She was managed with 100% oxygen and inotropes and cardiopulmonary resuscitation but despite aggressive medical interventions, her condition did not improve, and she unfortunately passed away. To diagnose, prevent, and manage the potentially devastating consequences associated with diagnostic hysteroscopy, gynecologists and surgical teams must maintain vigilance. The focus should be on proper patient selection, optimal surgical techniques, and the use of high-quality equipment to mitigate the risk of air embolism.

## Introduction

Diagnostic hysteroscopy is considered safer compared to operative hysteroscopy due to a lower incidence of adverse effects. Recently, gynecologists have widely adopted hysteroscopy as both a diagnostic and therapeutic tool for various gynecological issues such as infertility, fibroids, polyps, uterine cavity adhesions, uterine anomalies, and cervical pathology. It is essential to employ preventive measures, and understand and effectively manage potential complications associated with this procedure. Neglecting contraindications or using inappropriate surgical methods or instruments can lead to complications in hysteroscopy.

Studies have shown that subclinical air embolism may be present in all patients undergoing hysteroscopic procedures, with its incidence varying depending on the method of detection, ranging from 10% to 50% [1,2]. We recently encountered a rare case of air embolism that developed as a complication of diagnostic hysteroscopy in a young female undergoing a fertility work-up. In this report, we discuss strategies for prevention and management in future cases.

## Case presentation

A twenty-nine-year-old patient, married for 9 years with a history of various infertility treatments at outside hospitals. She was scheduled for diagnostic hysteroscopy and endometrial biopsy under general anesthesia as a part of her routine infertility workup following which the plan was to schedule her for embryo transfer (ET) as part of in vitro fertilization (IVF) treatment in our hospital. She had a history of two previous spontaneous abortions, one at one-and-a-half months (5 years ago) and the other at two-and-a-half months (4 years ago), followed by a recent IVF triplet conception (5 months ago). She underwent selective fetal reduction (3 months ago) for the triplet pregnancy, but both remaining twins developed oligohydramnios, leading to spontaneous abortion (2.5 months back) despite conservative measures. She also reported two failed intrauterine inseminations and two previous diagnostic laparoscopic procedures. There was no significant family history.

After conducting a thorough pre-anesthetic checkup (PAC), we induced general anesthesia by administering 3 mg/kg of propofol and 1 µg/kg of fentanyl intravenously (IV). We followed this with IV rocuronium at a dose of 0.6 mg/kg to facilitate endotracheal intubation using a laryngoscope. The anesthesia was maintained using isoflurane/O2/N2O. An infusion of remifentanil 0.1 µg/kg/min was given to maintain the intraoperative analgesia. The patient was placed in lithotomy with Trendelenburg position. The diagnostic hysteroscopy was performed after dilating the cervix, using normal saline as a distension media through the mechanical pump.

**Figure 1:**
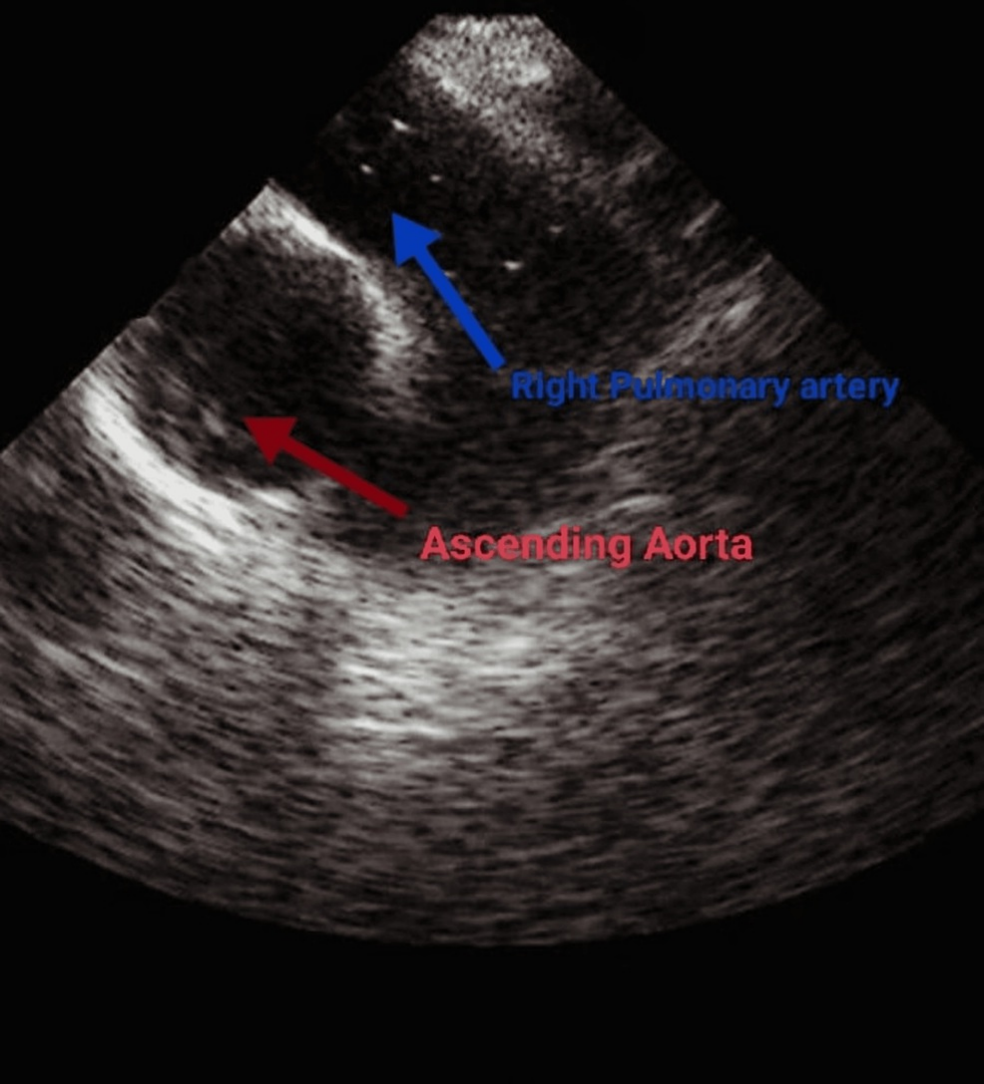
Transoesophageal echocardiography demonstrating air bubbles in the pulmonary artery.

Within 30 minutes of the procedure, the anesthesiologist observed a sudden decrease in oxygen saturation to 40%, along with a drop in end-tidal carbon dioxide (EtCO2) levels and an increase in heart rate to 150 bpm. This raised suspicion of an air embolism. The procedure was immediately stopped and she was instantly placed in the supine position. Quickly 100% O2, atropine (0.6 mg) and adrenaline (1 mg) were administered and cardiopulmonary resuscitation was started. She was intubated and dopamine infusion was initiated. Subsequently, SpO2 (peripheral oxygen saturation) and EtCO2 levels improved, and blood pressure rose to 90/60 mmHg with a pulse of 120 bpm. The ECG revealed sinus tachycardia and no other abnormality was detected. Transoesophageal echocardiography (TEE) was done which confirmed the presence of air bubbles in the right pulmonary artery as depicted in Figure 1.

The patient was transferred to ICU; she was put on inotropes, antibiotics, and dexamethasone. Despite proper intensive care in the ICU, her condition deteriorated further and she could not be revived on postoperative day 2. The postmortem examination of the patient could not be performed due to the lack of consent from the patient's relatives.

## Discussion

Etiopathogenesis

Air embolism is a risk factor associated with the use of carbon dioxide (CO2) as a distension medium in hysteroscopy. These risk factors include room air entry during cervical dilatation, the Trendelenburg position, or gaseous byproducts produced during electrosurgery, which are particularly common in operative hysteroscopy [3]. Room air, which is a mixture of oxygen and nitrogen, has a higher risk of developing an air embolism than carbon dioxide because CO2 is more soluble in blood than oxygen [4]. The fatal effects of an air or gas embolism are cardiac or pulmonary failure or death. The typical air or gas embolism symptoms are dyspnea and chest pain. If the patient is under anesthesia, an air embolism should be suspected if there is a drop in EtCO2 pressure or hypotension, or if hemodynamic abnormalities that resemble tachycardia are present. In the literature, very few cases of clinically severe gas embolism events are linked to hysteroscopic procedures [5,6]. Various risk factors are depicted in Table 1.

**Table 1:** Factors increasing the risk of air embolism. Source [3,4].

Risk Factors
1. The entry of room air during cervical dilatation or instrumentation,
2. Steep Trendelenburg position.
3. Gaseous byproducts of diathermy, particularly those observed during surgical hysteroscopy.
4. Compared to carbon dioxide, room air, a combination of nitrogen and oxygen is more commonly related to air embolism.

Preventive methods

The removal of air from hysteroscopic tools and in-flow tubing, restricting the continuous cervical application of instruments that can force air into the uterine cavity in a "piston-like" fashion, and lowering intrauterine pressure are all preventive strategies for air or gas embolism. Reduce cervical trauma to the absolute minimum, and, if necessary, use osmotic dilators before surgery. Keep the os closed at all times to prevent uterine air intrusion. As soon as the hysteroscope or resectoscope is fitted up, try to maintain the preceding dilator inside. The air embolism prevention checklist is shown in Table 2 [7,8,9].

**Table 2:** Important prerequisite for air embolism prevention. Source [7-10].

Air Embolism Prevention Checklist
The use of external pressure infusers should not be encouraged.
Fluid bottles should be kept at a height of < 1 meter above the patient.
Before every case change the tubing and hysteromat set to confirm that it is free of air bubbles. All connections must be checked in advance for the leak. Uterine distension medium fluid must be free of air bubbles. A single OT employee needs to be assigned to monitor for air bubbles and replace the tubing. Prefill fluid set tubing so that air bubble is avoided. Use mechanised pumps for distension and irrigation with fluid media. For changing the bottles Y-connector should be attached to the inflow line to prevent the air from entering in the system.
Echocardiography either a transthoracic (TTE) or a Transesophageal (TEE) probe can be employed if it is available. Since TEE is the most sensitive, it can also detect shunting from right to left and can spot gas bubbles as little as 0.5 cc.
Intrauterine pressure should be kept at <100 mmHg during the procedure.
Not to insert and remove the hysteroscope repeatedly.
The anesthesiologist should be informed to remain alert in case of any uterine injury as it increases the risk of embolism.
Confirm the availability of resuscitation equipment, central venous catheters, arterial cannulas and emergency inotropic drugs are ready prior to the procedure.
To lower the danger of an air embolism, the intracervical injection of vasopressin can be given.
Dilatation and curettage should be performed after hysteroscopy if they are to be performed in conjunction with it.

In the present case, the mechanized pump was employed for the procedure, the irrigation tubing was devoid of air bubbles and there was no repeated insertion of the hysteroscope into the uterine cavity. Before hysteroscopy dilatation of the cervical os was done with Hegar’s dilator instead of an osmotic dilator in the present case.

Treatment

Primary care for air embolism includes both passive and active interventions, such as an immediate cessation of the hysteroscopic procedure and deflation of the uterine cavity. By putting the patient in the left lateral decubitus position and Trendelenburg position, Durant’s manoeuvre can be carried out to assist in moving air toward the right ventricle to lessen the obstruction at the right ventricular outflow tract [4]. Table 3 depicts the management of air embolism.

**Table 3:** Management of air embolism Source [10]

Management of Air Embolism
An immediate halt to the hysteroscopic surgery and deflation of the uterine cavity.
Close the air entry point.
The woman can be placed in the Trendelenburg and left lateral decubitus positions to conduct Durant's manoeuvre, which helps move air in the direction of the right ventricle to lessen congestion at the right ventricular outflow tract.
100% O2 to be given, stop N2O.
Air removal should be considered via the insertion of a central venous catheter or direct cardiac aspiration.
Cardiac massage: to break large air bubbles.
Inotropic support and cardiopulmonary resuscitation (CPR).

In our case, upon clinical suspicion, we immediately halted the procedure. We administered all conservative measures, including 100% oxygen, inotropes, and cardiopulmonary resuscitation (CPR). However, we did not perform Durant's maneuver. Furthermore, following confirmation of air embolism via transesophageal echocardiography (TEE), consideration should have been given to aspirating air bubbles.

Verma and Singh in their study reported various cases of hysteroscopic myomectomy and discussed the ways to prevent, diagnose, and manage air embolism in reported three out of 13 such cases [10].

Girish and Singla (2016) from New Delhi, India reported a case of fatality in a case of diagnostic hysteroscopy despite timely intervention, which is similar to our case [11].

## Conclusions

Air embolism is an uncommon complication of diagnostic hysteroscopy. As the saying goes, 'Eyes can't see what the mind doesn't know.' Unfortunately, we learned this the hard way at the cost of a human life. It's essential to emphasize that managing an emergency like air embolism is a collaborative effort involving gynecologists, anesthesiologists, and operating room staff. When performing this seemingly simple procedure, surgeons must take all necessary preventive measures, be aware of the signs and symptoms of air embolism, and be prepared to respond promptly to save lives.
